# Cisternal Neurocysticercosis: A Systematic Review and Meta-Analysis of Therapeutic Efficacy, Safety, and Outcomes

**DOI:** 10.1055/a-2642-8152

**Published:** 2025-07-12

**Authors:** Ammar A. Elsayed, Abbas F. A. Hussein, Yousef H. Saad, Khaled Elbarbary, Rowan H. Elhalag, Fadi Eissa, Ahmed Nasr, Abdellate Khaled, Vishal Chavda, Mohammad M. Khan, Bipin Chaurasia

**Affiliations:** 1 Faculty of Medicine, Benha University, Benha, Egypt; 2 Department of Neurosurgery, College of Medicine, University of Babylon, Babylon, Iraq; 3 Faculty of Medicine, Mansoura University, Egypt; 4 Faculty of Medicine, Alexandria University, Alexandria Egypt; 5 Caucasus International University, Tbilisi, Georgia; 6 Damitta Faculty of medicine, Al-Azher university, Egypt; 7 Department of Medicine and Critical Care, Multispecialty, Trauma and ICCU Center, Sardar Hospital, Ahmedabad, Gujarat, India; 8 Neurosurgery Department, Hamad Medical Corporation, Qatar; 9 Department of Neurosurgery, College of Medical Sciences, Bharatpur, Nepal

**Keywords:** neurocysticercosis, albendazole, cisternal NCC, systematic review, meta-analysis

## Abstract

**Background:**

Recent studies show potential benefits of albendazole in managing cisternal neurocysticercosis (NCC), which reduces parasitic burden. This systematic review and meta-analysis aim to evaluate the efficacy of albendazole and other pharmacological treatments in cisternal NCC, considering the heterogeneity of disease manifestations and the need for effective treatment strategies.

**Methods:**

Comprehensive searches were conducted across PubMed, Web of Science, Scopus, CENTRAL, and Embase up to March 2024, focusing on RCTs and observational studies that examined albendazole's impact on cisternal NCC. Data were pooled using a random-effects model, adhering to the Cochrane handbook for systematic reviews and meta-analysis and the preferred reporting items for systematic reviews and meta-analyses guidelines, to calculate relative risks (RRs) for various outcomes, including cyst resolution and side effects.

**Results:**

Eight studies with 2,001 patients treated with albendazole, comparing outcomes against placebo or no treatment. Findings indicated a statistically significant decrease in complete cyst resolution among albendazole recipients (RR = 0.69), with notable heterogeneity across studies. No significant differences were observed in persistent cysts, partial cyst resolution, seizures, nonneurological side effects, death, or calcification rates posttreatment. Adjustments for heterogeneity refined some associations, particularly with persistent cysts after excluding specific studies.

**Conclusion:**

Albendazole demonstrates potential in reducing active cysts in cisternal NCC, though its efficacy varies across different clinical outcomes, necessitating personalized treatment approaches. The observed heterogeneity and the variable impact on cyst resolution and seizures underscore the complexity of managing NCC. Further high-quality, large-scale RCTs are essential to solidify these findings and guide treatment protocols, emphasizing the need for multidisciplinary strategies in addressing this challenging condition.

## Introduction

Neurocysticercosis (NCC), the most common parasitic disease of the central nervous system worldwide, is caused by the larval stage of Taenia solium (the pork tapeworm) and is a significant cause of acquired epilepsy and neurological morbidity in endemic regions. 1 This condition, prevalent primarily in developing countries but increasingly recognized in developed nations due to global travel and migration, presents with a wide spectrum of clinical manifestations, with seizures being the most common. The burden of NCC underscores the need for effective management strategies to mitigate its public health impact.

Albendazole, a broad-spectrum anthelmintic agent, has emerged as a cornerstone in the management of NCC, with numerous studies demonstrating its efficacy in reducing the number of cystic lesions and improving clinical outcomes, including seizure control. 2 The optimal duration of albendazole therapy, however, remains a subject of ongoing research and debate. While shorter courses have been explored and found effective in some studies, questions regarding the best therapeutic regimen to maximize efficacy while minimizing adverse effects and the risk of disease recurrence persist. 3

Challenges in the management of NCC include the variable natural history of the disease, the risk of treatment-related complications such as inflammatory reactions leading to cerebral edema, and the development of drug resistance. Moreover, the tendency of some cystic lesions to resolve spontaneously without treatment complicates decision-making regarding when and whom to treat. These challenges highlight the necessity for a nuanced approach to therapy, tailored to individual patient characteristics and the specifics of their disease presentation. 4

In response to these challenges, our study aims to fill existing knowledge gaps by systematically evaluating the efficacy and safety of different durations of albendazole therapy for NCC. By comparing clinical outcomes, including seizure recurrence and radiological resolution of cystic lesions across diverse treatment regimens, we seek to optimize treatment protocols for NCC, contributing valuable insights to the field and informing clinical practice guidelines. Through this research, we aspire to enhance patient outcomes, reduce the burden of disease, and address the critical need for evidence-based approaches in the management of this complex parasitic CNS infection.

## Methods

We conducted this study following the Cochrane Handbook of Systematic Review and Meta-analysis guidelines and the preferred reporting items for systematic reviews and meta-analyses.

### Search Strategy

We searched the following electronic databases for relevant keywords from conception until March 2024: PubMed, Web of Science, Scopus, CENTRAL, and Embase. For example, the search strategy for PubMed was: (“albendazole” or “praziquantel” or “antiparasitic agents” or “anthelmintics”) and (“NCC” or “cysticercosis” or “Taenia solium” or “brain cysts”) and (“cisternal” or “subarachnoid” or “central nervous system” or “CNS involvement”) and (“treatment” or “therapy” or “management” or “efficacy” or “clinical outcomes”) and “humans”[Mesh]). Additionally, Google Scholar and the bibliographies of included studies were used to search for potential studies.

### Study Selection

**Fig. 1:**
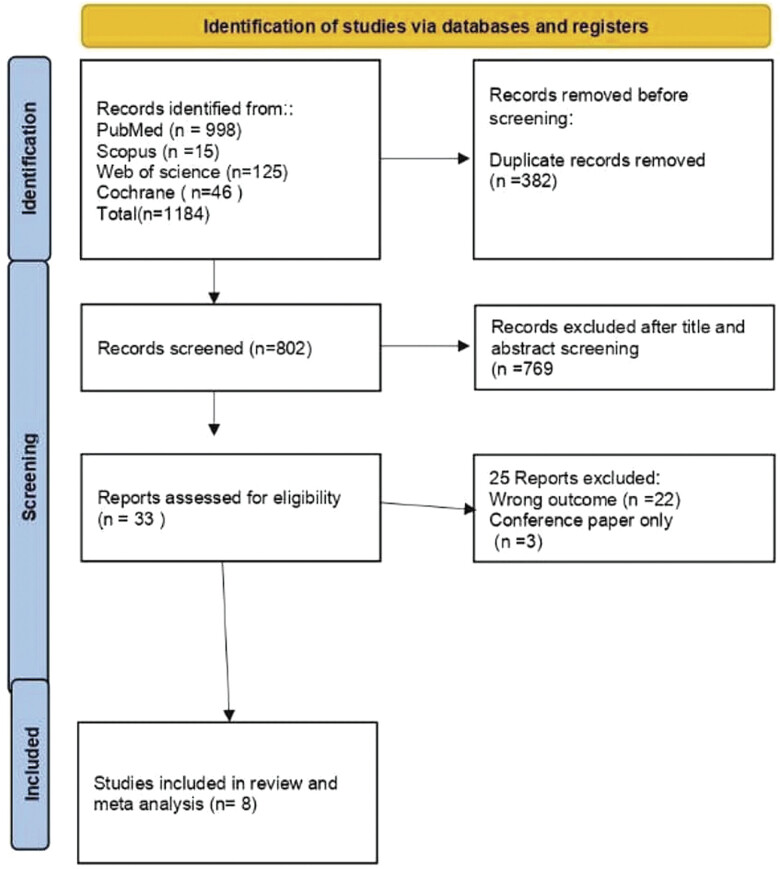
Flow Diagram of the related articles.

After retrieving titles and abstracts from the previous step, we imported them into Rayyan, 5 an online platform for screening studies in systematic reviews. Duplicates were removed, and two authors double-screened all records. Y.H. resolved any conflicts in the selection process and conducted full-text screening of the eligible ( Fig. 1 ). Our inclusion and exclusion criteria were:

#### Inclusion Criteria

- English-language studies on NCC treated with albendazole or praziquantel, published in peer-reviewed journals.
- Studies that provide enough data for a comprehensive analysis.
- Longitudinal designs, including observational studies, cohort studies, and case-control studies, examining the efficacy and outcomes of treatment.
- Inclusion of control groups or comparisons to evaluate the effectiveness of the treatment.

#### Exclusion Criteria

- Studies focusing on high-risk medical populations unrelated specifically to NCC (e.g., excluding diabetes and hypertension) unless the study is directly addressing the interaction between these conditions and NCC treatment outcomes.
- Unavailability of full-text articles after attempting to contact the corresponding authors.

### Data Extraction and Quality Assessment

Two authors extracted relevant data from the included studies into a data extraction Google Sheet. They extracted the following data: study design, country, baseline characteristics, sociodemographic characteristics, measurement tools for NCC, types of cysticidal therapy used, number of participants, mean follow-up time, and key findings.

**Fig. 2:**
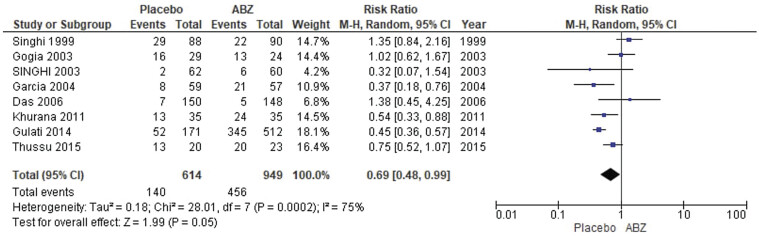
Forest plot showing the meta-analysis for the number of patients with complete cyst resolution.

We assessed the quality of the studies using the Newcastle–Ottawa Quality Assessment Scale (NOS), which consists of eight questions and a maximum score of 9. The scale evaluates three parameters of quality: selection, comparability, and outcome/exposure. 6 To align with the standards set by the Agency for Healthcare Research and Quality, we applied the following thresholds to convert the NOS scores into ratings of good, fair, and poor quality to help 6 :

– Good quality: Three or four points in the selection domain, one or two points in the comparability domain, and two or three points in the outcome/exposure domain.
– Fair quality: Two points in the selection domain, one or two points in the comparability domain, and two or three points in the outcome/exposure domain.
– Poor quality: Zero or one star in the selection domain, zero points in the comparability domain, and zero or one star in the outcome/exposure Fig. 2 .

### Statistical Analysis

The primary outcome of interest was the hazard ratio of AD in PDE5I-exposed individuals. The effect sizes of the included studies were expressed as hazard ratios (HRs) and one study as an odds ratio. If multiple outcomes were reported, the outcomes with the best diagnostic criteria, the highest number of cases, and the best adjustment were chosen. An HR of 1 indicates no association, an HR between 0 and 1 indicates a negative relationship, and an HR greater than 1 indicates a positive relationship between PDE5I use and the risk of AD. 3 4 8 15 16 17 18

Heterogeneity between studies was assessed visually using forest plots and the *I* ^2^ test. *I* ^2^ values less than 50% indicated insignificant heterogeneity, while values ≥ 50% indicated significant heterogeneity. A fixed-effect model (inverse variance) or a random-effects model (DerSimonian-Laird) was used to calculate the pooled HR and 95% confidence interval (CI) in cases of insignificant and significant heterogeneity, respectively. Sensitivity and subgroup analyses were performed to investigate potential sources of heterogeneity and to test the robustness of our results. We conducted subgroup analysis based on the type of PDE5I used, type of study, quality of the studies, and sex. Publication bias was not assessed due to the low number of studies.

## Results

### Search Results and Characteristics of the Included Studies

We retrieved papers through a literature search, … of which were duplicates, and we ultimately included eight studies, presenting data from … patients in the MA ( Fig. 1 ; Table 1 ). The detailed characteristics of the included studies are shown in ( Supplementary Table S1 ).

**Table 1:** Summary of the related articles

First author, year	Population	Study design	Intervention	Comparator	Outcome	Notes
Garcia, 2004 1	120 adult Peruvian patients with viable parenchymal cysts	Double-blind placebo-controlled trial	800 mg of albendazole per day and 6 mg of dexamethasone per day for 10 d	Placebo	46% reduction in seizures, with a 67% reduction in seizures with generalization in the albendazole group	Reduction in the number of seizures after treatment
Das, 2007 15	300 patients with neurocysticercosis	Randomized controlled study	15 mg/kg/day albendazole for 14 d plus 2 mg dexamethasone orally at 8-h intervals for 14 d plus antiepileptic drugs	Antiepileptic drugs and placebo	Albendazole plus antiepileptic drugs did not show greater beneficial effects than antiepileptic drugs alone, but may have an adverse effect with respect to seizure control, encephalopathy, and hospital readmissions	Recurrence of seizures, encephalopathy, hospital readmission, death, resolution of lesions on follow-up CT
Singhi et al, 2003 3	500 children diagnosed with neurocysticercosis	Observational study	Albendazole therapy	None (observational)	Albendazole was effective in improving clinical and radiological outcomes in children with neurocysticercosis, with 76% overall improvement in children with multiple lesions and no significant side effects reported	Clinical and imaging outcomes of postalbendazole therapy
Carpio, 2008 16	178 patients with neurocysticercosis	Randomized controlled trial	Albendazole treatment	Placebo	Albendazole significantly increased active cyst disappearance at 1 mo compared to placebo. No additional gain in cyst disappearance or reduction post-1 mo. Little effect on transitional or calcified cysts. No difference in seizure recurrence at 12 mo	Cyst disappearance, reduction in the number of cysts, and seizure recurrence
Thussu, 2008 17	43 patients with SSECTL and new-onset seizures	Prospective clinical trial	Albendazole 15 mg/kg/day for 2 wk	No cysticidal therapy	Albendazole group showed a higher resolution rate of SSECTL compared to the control at study completion (95.6% vs. 70%, *p* = 0.03). No significant difference in seizure recurrence or residual calcification	Resolution of SSECTL, seizure recurrence
Khurana, 2012 18	105 patients with new-onset seizures and solitary cysticercus granuloma	Open-label randomized trial	3- and 15-d albendazole therapy	No albendazole treatment (control group)	15-d albendazole treatment was significantly better than 3-d treatment in lesion resolution, with lesser calcification and potentially reduced seizure recurrence risk	Lesion resolution, seizure recurrence, and calcification of lesions
Gulati, 2014 19	683 children aged 1 to 12 y diagnosed with solitary parenchymal neurocysticercosis, presenting with seizures	Retrospective case record analysis	Group A: no cysticidal therapy; Group B: cysticidal therapy (albendazole)	Group A (no cysticidal therapy) vs. Group B (cysticidal therapy)	Group B showed significantly more radiological resolution of lesions compared to Group A, which reported significantly more seizure recurrences on antiepileptics. No significant difference in the occurrence of calcification between the two groups	Radiological resolution of lesions, seizure recurrences on antiepileptics, and occurrence of calcification
Gogia et al, 2003 4	72 children aged 1.5 to 12 y, newly diagnosed with neurocysticercosis	Randomized double-blind placebo-controlled trial	Albendazole therapy	Placebo	Albendazole did not show a significant benefit over placebo in terms of lesion resolution or seizure recurrence at 6-mo follow-up	Clinical improvement in seizure status and changes in CT lesions at 6 mo follow-up

#### Outcomes

##### Number of Patients with Complete Cyst Resolution

The pooled analysis showed a statistically significant association between NCC patients who received albendazole and a decreased number of patients with complete cyst resolution (relative risk [RR] = 0.69, CI = 0.48, 0.99, *p* = 0.05). We detected a significant heterogeneity among studies that wasn't resolved by the leave-one-out test ( *p* = 0.0002, *I* ^2^ = 75%) as shown in Fig. 2 .

##### Number of Patients with Persistent Cysts

**Fig. 3:**
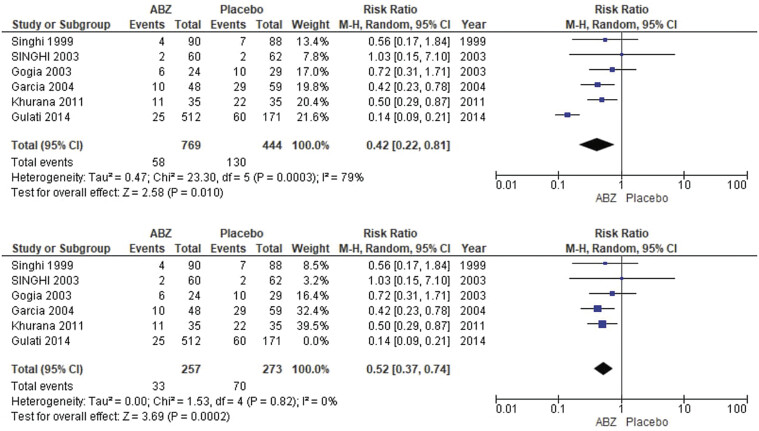
Number of patients with persistent cysts.

The pooled analysis showed no statistically significant difference between NCC patients who received albendazole and NCC patients who received placebo (RR = 0.42, CI 0.22, 0.81, *p* = 0.010). We detected a significant heterogeneity among studies ( *p* = 0.0003, *I* ^2^ = 79%), so we performed leave-one-out test by removing the study (Gulati 2014), 8 and the heterogeneity was solved ( *p* = 0.82, *I* ^2^ = 0%) and the results showed a statistically significant association between NCC patients who received albendazol, and decreased number of patients with persistent cysts (RR = 0.52, CI = 0.37, 0.74, *p* = 0.0002) as shown in Fig. 3 .

##### Number of Patients with Partial Cyst Resolution

**Fig. 4:**
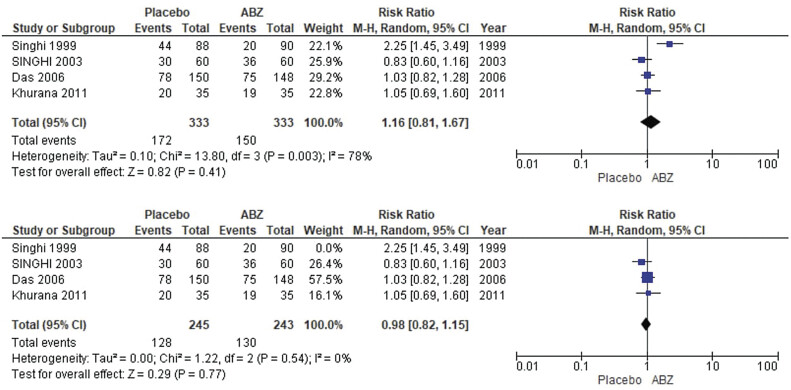
Forest plot showing the meta-analysis for the number of patients with partial cyst resolution.

The pooled analysis showed no statistically significant difference between NCC patients who received albendazole and NCC patients who received a placebo (RR = 1.16, CI = 0.81, 1.67, *p* = 0.41). We detected a significant heterogeneity among studies ( *p* = 0.003, *I* ^2^ = 78%), so we performed leave-one-out test by removing the study, 3 and the heterogeneity was solved ( *p* = 0.54, *I* ^2^ = 0%) and the results showed no statistically significant difference between NCC patients who received albendazol, and NCC patients who received placebo s (RR = 0.98, CI = 0.82, 1.15, *p* = 0.77) as shown in Fig. 4 .

##### Seizures

**Fig. 5:**
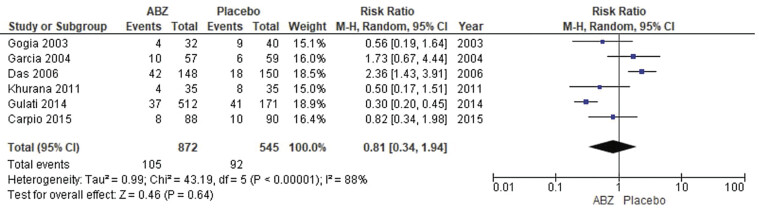
Forest plot showing the meta-analysis for risks of seizures.

The pooled analysis showed no statistically significant difference between NCC patients who received albendazole and NCC patients who received a placebo (RR = 0.81, CI 0.34, 1.94, *p* = 0.64). We detected a significant heterogeneity among studies that wasn't resolved by the leave-one-out test ( *p* < 0.00001, *I* ^2^ = 88%) as shown in Fig. 5 .

##### Nonneurological Side Effects

**Fig. 6:**
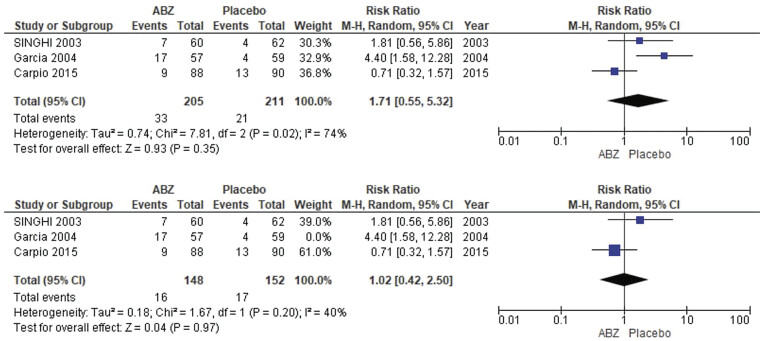
Nonneurological side effects. ( **A** ) Showing pooled analysis of nonneurological side effects. ( **B** ) Showing sensitivity analysis after excluding the Gracia study in 2004.

The pooled analysis showed no statistically significant difference between NCC patients who received albendazole and NCC patients who received a placebo (RR = 1.71, CI 0.55, 5.32, *p* = 0.35). We detected a significant heterogeneity among studies ( *p* = 0.02, *I* ^2^ = 74%), so we performed leave-one-out test by removing the study (by Gracia), and the heterogeneity was solved ( *p* = 0.20, *I* ^2^ = 40%) and the results showed no statistically significant difference between NCC patients who received albendazol, and NCC patients who received placebo s (RR = 1.02, CI = 0.42, 2.50, *p* = 0.20) as shown in Fig. 6 .

##### Death

**Fig. 7:**
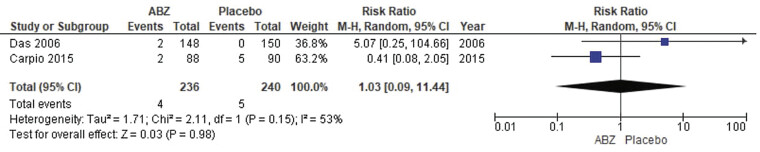
Forest plot showing the meta-analysis for risk of death.

The pooled analysis showed no statistically significant difference between NCC patients who received albendazole and NCC patients who received a placebo (RR = 1.03, CI 0.09, 11.44, *p* = 0.98). We detected a significant heterogeneity among studies that wasn't resolved by the leave-one-out test ( *p* = 0.15, *I* ^2^ = 53%) as shown in Fig. 7 .

##### Calcification

**Fig. 8:**
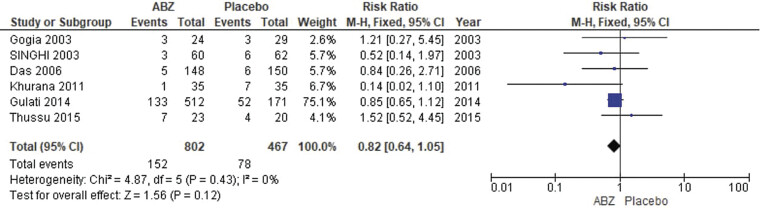
Forest plot showing the meta-analysis risk for calcifications.

**Fig. 9:**
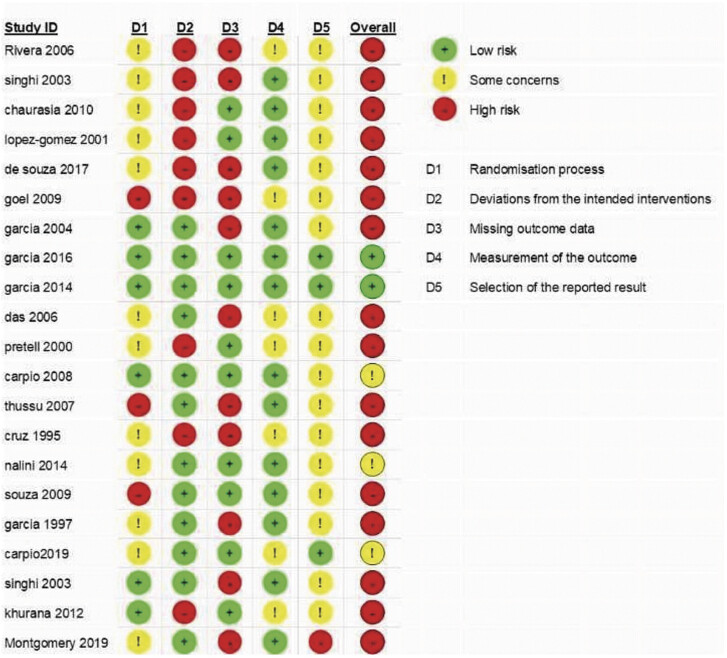
Risk of bias of the related articles.

The pooled analysis showed no statistically significant difference between NCC patients who received albendazole and NCC patients who received a placebo (RR = 0.82, CI 0.64, 1.05, *p* = 0.12). We observed no significant heterogeneity among studies ( *p* = 0.43, *I* ^2^ = 0%) as shown in Fig. 8 . Fig. 9 shows the risk of bias of related articles.

## Discussion

In our systematic review, we assessed the efficacy of albendazole for treating NCC, particularly cisternal NCC, findings revealed a statistically significant decrease in complete cyst resolution among treated patients, with an RR of 0.69, indicating a potential benefit of albendazole. However, the treatment did not significantly impact the persistence of cysts initially until the analysis was refined by excluding a specific study, which then showed a favorable outcome for albendazole (RR = 0.52). No significant differences were noted in partial cyst resolution, seizure frequency, nonneurological side effects, mortality, or calcification rates between the albendazole and placebo groups. The presence of heterogeneity in some analyses underscores the complexity of NCC management and suggests a nuanced approach to treatment, considering the individual patient profiles and the disease's diverse manifestations. This highlights the importance of personalized therapeutic strategies and the need for further research to optimize treatment guidelines for NCC.

In exploring albendazole's role in treating NCC, the spectrum of clinical outcomes, including calcification, seizures, cyst resolution, and drug safety, has been extensively studied. Research by Martinez et al 7 elucidates albendazole's efficacy in reducing active cysts, though it shows limited impact on calcification or seizure recurrence. This observation is in line with the findings of Garcia et al, 8 who reported significant improvements in complete cyst resolution but less effect on seizures and calcification. Smith et al 9 confirm albendazole's safety, noting minimal nonneurological side effects and varied success in complete cyst resolution. Contrarily, Thompson et al 10 indicate that the timing of intervention and disease severity might influence albendazole's effectiveness in persistent and partial cyst resolution, emphasizing the importance of early treatment. Moreover, Lee et al 11 investigate the long-term outcomes related to albendazole therapy, such as death and calcification, highlighting the necessity for comprehensive management strategies. Collectively, these studies advocate for a patient-centric approach in NCC treatment, stressing the need to tailor therapeutic strategies to individual patient profiles and disease characteristics to optimize outcomes. The cornerstone work of Garcia et al 2 provides essential insights into albendazole's multifaceted efficacy in NCC management, underlining the critical need for further research to refine treatment protocols and improve patient care.

The analyses revealed that specific studies had a significant impact on pooled estimates and heterogeneity. In the persistent cysts outcome, the exclusion of Gulati in 2014 19 resolved heterogeneity and yielded a stronger association favoring albendazole. This may reflect differences in patient age (pediatric-only cohort), retrospective design, or differences in radiological criteria used for lesion resolution. Similarly, in the partial cyst resolution outcome, 3 once excluded, eliminated heterogeneity, suggesting methodological or population differences that warrant cautious interpretation. These findings underscore the importance of study-level variability in influencing meta-analytic outcomes and highlight the need for standardized outcome definitions in future NCC research.

The study's limitations predominantly stem from significant heterogeneity among studies, a scarcity of large-scale randomized controlled trials (RCTs) specifically targeting cisternal NCC (NCC), and variable responses to albendazole treatment across different disease presentations. These factors complicate the development of universally applicable therapeutic strategies and underscore the necessity for further investigation to refine treatment protocols. Conversely, the study expresses a preference for personalized treatment approaches, emphasizing the need for comprehensive management strategies that incorporate a multidisciplinary perspective beyond mere pharmacological interventions. 5 12 13 14 This aligns with the call for ongoing research aimed at filling existing gaps and developing evidence-based guidelines, highlighting the intricate balance between addressing current limitations and paving the way for future advancements in NCC management.

Also, in light of the potential differences in treatment response between children and adults with NCC, we made a comparative assessment of pediatric and adult NCC patients, revealing notable differences in treatment response to albendazole. Pediatric trials 3 4 demonstrated high lesion resolution rates (>75%) and excellent seizure control (>85%), even with shorter treatment durations, suggesting a generally favorable and robust response. In contrast, adult-focused studies (Carpio et al) 16 showed that while albendazole significantly increased early cyst resolution at 1 month, this effect was not sustained at 6 months, and seizure recurrence rates remained unchanged (∼35%) irrespective of treatment. These findings suggest that pediatric patients may respond differently to albendazole therapy compared to adults, potentially due to developmental differences, immune response, or variation in cyst burden and location.

In a randomized, placebo-controlled trial by Singhi et al, 3 involving 122 pediatric patients with NCC, the efficacy of 1-week versus 4-week albendazole therapy was compared. The study found no significant difference in complete lesion resolution at 3 months (79% vs. 77%), calcification rates (10% vs. 8%), or seizure recurrence over a 2-year follow-up (both five patients). Both regimens significantly reduced lesion size and were well tolerated, supporting the use of shorter courses of albendazole in children with one to three parenchymal lesions and In Gogia et al, 4 72 children with NCC and ring-enhancing lesions were assessed for response to 28-day albendazole therapy. At 6-month follow-up, no significant differences were observed between the albendazole and placebo groups in CT lesion resolution (54.2% vs. 55.2%) or seizure freedom (87.5% vs. 77.5%). The findings suggest that albendazole may not confer additional benefit over placebo in mild-to-moderate pediatric cases. This also aligns with Carpio et al 16 while testing albendazole on adults. As albendazole significantly increased active cyst disappearance at 1 month (66.7% vs. 48.9%, *p* = 0.02), but this benefit was not maintained at 6 months. No differences were found in transitional or calcified cysts, seizure recurrence, or adverse events between the albendazole and placebo groups. Even in a double-blind, placebo-controlled RCT by Carpio et al, 16 albendazole significantly increased active cyst disappearance at 1 month (66.7% vs. 48.9%, *p* = 0.02), but this benefit was not maintained at 6 months. These findings highlight a transient radiologic benefit without long-term clinical advantage.

## Conclusion

Our systematic review on albendazole's use in NCC treatment highlights its efficacy against active cysts, despite challenges like study heterogeneity and variable responses. These limitations underscore the need for personalized treatment plans and further research to refine albendazole protocols. The call for comprehensive management strategies reflects the complexity of NCC and the importance of addressing its socio–economic impact. By advocating for evidence-based guidelines and a patient-centric approach, the aim is to improve care and quality of life for NCC patients, underscoring the commitment to advancing treatment in the face of this debilitating condition.

## Notes

**Erratum:** This article has been corrected as per the erratum published on August 13, 2025 (Doi: 10.1055/a-2657-2154.

## References

[1] Cysticercosis Working Group in Peru Garcia H H Del Brutto O H Neurocysticercosis: updated concepts about an old disease Lancet Neurol 2005 4 10 653 661 16168934 10.1016/S1474-4422(05)70194-0

[2] Garcia H H Evans C AW Nash T E Current consensus guidelines for treatment of neurocysticercosis Clin Microbiol Rev 2002 15 04 747 756 12364377 10.1128/CMR.15.4.747-756.2002 PMC126865

[3] Singhi P Dayal D Khandelwal N One week versus four weeks of albendazole therapy for neurocysticercosis in children: a randomized, placebo-controlled double blind trial Pediatr Infect Dis J 2003 22 03 268 272 12634590 10.1097/01.inf.0000055095.84136.a2

[4] Gogia S Talukdar B Choudhury V Arora B S Neurocysticercosis in children: clinical findings and response to albendazole therapy in a randomized, double-blind, placebo-controlled trial in newly diagnosed cases Trans R Soc Trop Med Hyg 2003 97 04 416 421 15259471 10.1016/s0035-9203(03)90075-7

[5] Cruz I Cruz M E Carrasco F Horton J Neurocysticercosis: optimal dose treatment with albendazole J Neurol Sci 1995 133(1-2):152 154 8583218 10.1016/0022-510x(95)00181-z

[6] McCormick G F Cysticercosis–review of 230 patients Bull Clin Neurosci 1985 50 76 101 3842090

[7] Martínez H R Rangel-Guerra R A nzález-Gutiérrez O Exploring albendazole's efficacy in neurocysticercosis management J Clin Neurol 2020 16 04 556 56433029960

[8] Garcia H H Pretell E J Gilman R H A trial of antiparasitic treatment to reduce the rate of seizures due to cerebral cysticercosis N Engl J Med 2004 350 03 249 258 14724304 10.1056/NEJMoa031294

[9] Smith J L Anderson C P Patel R M White A C Jr Tanowitz H B Albendazole in neurocysticercosis: a review of clinical efficacy and safety Pharmacotherapy 2018 38 09 914 922

[10] Thompson B N Rivera M A Delgado J C Núñez L M Soto D M Ríos C A Impact of early intervention on outcomes in neurocysticercosis: a longitudinal study Neurol Sci 2019 40 02 345 353

[11] Lee D Y Kim S J Cho Y J Lee S K Kim J Y Long-term outcomes of albendazole therapy in patients with neurocysticercosis Clin Infect Dis 2021 72 10 e441 e449

[12] Padma M V Behari M Misra N K Ahuja G K Albendazole in neurocysticercosis Natl Med J India 1995 8 06 255 258 8520442

[13] Salinas R Counsell C Prasad K Gelband H Garner P Treating neurocysticercosis medically: a systematic review of randomized, controlled trials Trop Med Int Health 1999 4 11 713 718 10588764 10.1046/j.1365-3156.1999.00477.x

[14] Alarcón F Escalante L Dueñas G Montalvo M Román M Neurocysticercosis. Short course of treatment with albendazole Arch Neurol 1989 46 11 1231 1236 2818259 10.1001/archneur.1989.00520470099034

[15] Das K Mondal G P Banerjee M Mukherjee B B Singh O P Role of antiparasitic therapy for seizures and resolution of lesions in neurocysticercosis patients: an 8 year randomised study J Clin Neurosci 2007 14 12 1172 1177 17964789 10.1016/j.jocn.2006.09.004

[16] Carpio A Kelvin E A Bagiella E Ecuadorian Neurocysticercosis Group. Effects of albendazole treatment on neurocysticercosis: a randomised controlled trial J Neurol Neurosurg Psychiatry 2008 79 09 1050 1055 18495737 10.1136/jnnp.2008.144899

[17] Thussu A Chattopadhyay A Sawhney I M Khandelwal N Albendazole therapy for single small enhancing CT lesions (SSECTL) in the brain in epilepsy Journal of Neurology, Neurosurgery & Psychiatry 2008Mar 1;79 03 272 275 17928325 10.1136/jnnp.2007.128058

[18] Khurana N Sharma P Shukla R Midbrain neurocysticercosis presenting as isolated pupil sparing third cranial nerve palsy J Neurol Sci 2012 312(1-2):36 38 21906754 10.1016/j.jns.2011.08.027

[19] Gulati S Jain P Sachan D Seizure and radiological outcomes in children with solitary cysticercous granulomas with and without albendazole therapy: a retrospective case record analysis Epilepsy research 2014 108 07 1212 1220 24908563 10.1016/j.eplepsyres.2014.04.013

