# Erratum: Cisternal Neurocysticercosis: A Systematic Review and Meta-Analysis of Therapeutic Efficacy, Safety, and Outcomes

**DOI:** 10.1055/a-2657-2154

**Published:** 2025-08-13

**Authors:** Ammar A. Elsayed, Abbas F. A. Hussein, Yousef H. Saad, Khaled Elbarbary, Rowan H. Elhalag, Fadi Eissa, Ahmed Nasr, Abdellate Khaled, Vishal Chavda, Mohammad M. Khan, Bipin Chaurasia

**Affiliations:** 1 Faculty of Medicine, Benha University, Benha, Egypt; 2 Department of Neurosurgery, College of Medicine, University of Babylon, Babylon, Iraq; 3 Faculty of Medicine, Mansoura University, Egypt; 4 Faculty of Medicine, Alexandria University, Alexandria Egypt; 5 Caucasus International University, Tbilisi, Georgia; 6 Damitta Faculty of medicine, Al-Azher university, Egypt; 7 Department of Medicine and Critical Care, Multispecialty, Trauma and ICCU Center, Sardar Hospital, Ahmedabad, Gujarat, India; 8 Neurosurgery Department, Hamad Medical Corporation, Qatar; 9 Department of Neurosurgery, College of Medical Sciences, Bharatpur, Nepal

It has been brought to the publisher's attention that the name of the author, Khaled Elbarbary, was incorrectly listed in an article published in *Journal of Neurological Surgery Reports* , Volume 86, Issue 3, e140–e148, on July 12, 2025 (doi:  ). This error has now been corrected in the author's byline. The correct author name also appears above.

