# Supplementary material for: Cisternal Neurocysticercosis: A Systematic Review and Meta-Analysis of Therapeutic Efficacy, Safety, and Outcomes
Source: J Neurol Surg Rep. 2025 Jul 12;86(3):e140–8. doi: 10.1055/a-2642-8152 (PMC12255487; doi:10.1055/a-2642-8152)
Supplement: Supplementary file 1 — Supplementary Material [file 10-1055-a-2642-8152_26544801.pdf]

Supplementary Table S1 The characteristics of the included studies

First author, year	Age (mean)	Steroid use	Treatment dose	Follow-up	Outcomes
Garcia, 2004		Dexamethasone per day for 10 days	800 mg	1–30 mo	46% reduction in seizures, with a 67% reduction in seizures with generalization in the albendazole group
Das, 2007		2 mg dexamethasone orally at 8-h intervals for 14 days			Recurrence of seizures, encephalopathy, hospital readmission, death, resolution of lesions on follow-up CT
Singhi, 2000					76% overall improvement in children with multiple lesions and no significant side effects reported
Carpio, 2008			1 mg		Cyst disappearance, reduction in the number of cysts, seizure recurrence
Thussu, 2008	24 year		18 mg	1–3 mo	Resolution of SSECTL, seizure recurrence
Khurana, 2012	17.14 year		15 mg	3–15 d	Lesion resolution, seizure recurrence, calcification of lesions. Lesser calcification and potentially reduced seizure recurrence risk
Gulati, 2014	7.9 year		15 mg		Radiological resolution of lesions, seizure recurrences on antiepileptics, occurrence of calcification
Gogia, 2003					Clinical improvement in seizure status and changes in CT lesions at 6 months follow-up

 © 2025. The Author(s).
